# Novel Quinazoline Derivative Induces Differentiation of Keratinocytes and Enhances Skin Barrier Functions against Th2 Cytokine-Mediated Signaling

**DOI:** 10.3390/molecules28166119

**Published:** 2023-08-18

**Authors:** Yukyung Park, Huddar Srigouri, Dongwon Kim

**Affiliations:** 1 Department of Energy and Biotechnology, Graduate School, Dongseo University, Busan 47011, Republic of Korea; 2 Drug Information Platform Center, Korea Research Institute of Chemical Technology, Daejeon 34114, Republic of Korea; srigouri@krict.re.kr; 3 Department of Bio-Pharmaceutical Engineering, College of Bio-Health Convergence, Dongseo University, Busan 47011, Republic of Korea

**Keywords:** atopic dermatitis, quinazoline, filaggrin, TSLP, STAT6

## Abstract

Atopic dermatitis (AD) is a common inflammatory skin disease characterized by pruritic lesions and skin barrier dysfunction. In this study, we evaluated the effect of a quinazoline derivative, SH-340, on TSLP expression and signaling in human primary keratinocytes. Our results demonstrated that SH-340 significantly increased factors for differentiation and skin barrier function including KRT1, KRT2, KRT10, IVL, LOR, CLDN1, OVOL1, and FLG, whereas it inhibited TSLP expression in a dose-dependent manner, both at the mRNA and protein levels. Furthermore, SH-340 was found to inhibit the phosphorylation of STAT6, a downstream signaling molecule of IL-4 and IL-13, in keratinocytes. These findings suggest that SH-340 may suppress TSLP expression by inhibiting the IL-4/IL-13-STAT6 signaling pathway. Finally, SH-340 may potentially contribute to both the alleviation of inflammation and the restoration of skin barrier function.

## 1. Introduction

Quinazolines are a class of heterocyclic compounds that have been extensively studied in the field of medicinal chemistry due to their wide range of biological activities. These compounds have been found to exhibit a variety of pharmacological activities, including anticancer, antimicrobial, and anti-inflammatory activities [1,2]. Previous studies support the biological activities of quinazolines in skin research. Studies have demonstrated that quinazolines show potent anti-inflammatory effects by inhibiting pro-inflammatory cytokines such as tumor necrosis factor-alpha (TNF-α), interleukin (IL)-1 beta, and IL-6 [3]. In addition, quinazolines inhibit the proliferation of skin cells and modulate the immune response in the skin [4,5]. Additionally, quinazolines have shown promising effects in promoting wound healing by stimulating fibroblast proliferation, collagen synthesis, and angiogenesis [6]. Furthermore, quinazolines have shown notable antioxidant activity, effectively scavenging reactive oxygen species (ROS) and protecting skin cells from oxidative stress-induced damage [7]. Some quinazoline derivatives have potential antimicrobial effects against common skin pathogens including *Staphylococcus aureus* [5,8]. Collectively, these findings highlight the significant potential of quinazolines in various aspects of skin research associated with inflammation modulation, wound healing, antioxidant protection, and antimicrobial applications.

Atopic dermatitis (AD) is characterized by chronic inflammation and a defective skin barrier, which allows for the penetration of irritants and allergens [9,10,11]. In AD patients, the skin barrier is weakened due to defects in the filaggrin (FLG) gene, which codes for a protein that helps to maintain the structural integrity of the stratum corneum, the outermost layer of the skin [10,12]. Overall, the regulation of the FLG gene involves a complex interplay of signaling mechanisms, transcription factors, cytokines, and genetic variations [11,12,13]. Accumulating evidence strongly supports that T help type 2 (Th2)-mediated cytokines (IL-4 and IL-13) have been implicated in the regulation of FLG gene expression by activating JAK (Janus kinase)-STAT signaling pathway [10]. Thus, current therapeutic strategies for AD aim to restore and maintain skin barrier function by inducing FLG expression, as well as reducing inflammation and pruritus. JAK inhibitors work by blocking the activity of JAKs, which leads to the activation of immune cells and the production of pro-inflammatory cytokines.

Several JAK inhibitors are currently being investigated for the treatment of AD, including baricitinib, upadacitinib, and abrocitinib [14,15]. Clinical studies have demonstrated that these JAK inhibitors can improve the signs and symptoms of AD, including reducing itching, redness, and skin lesions [16,17]. Recently, a quinazoline derivative called JTE-052 (delgocitinib) has been shown to reduce inflammation and improve skin barrier function in a mouse model of atopic dermatitis [18]. JTE-052 was found to inhibit the production of pro-inflammatory cytokines and chemokines such as TNF-α, IL-1β, IL-6, and CCL17 in the skin. It also decreased the infiltration of inflammatory cells such as eosinophils and mast cells into the skin. In addition, JTE-052 was found to increase the expression of proteins that are involved in maintaining the integrity of the skin barrier, such as loricrin (LOR), involucrin (IVL), and FLG.

**Figure 1:**
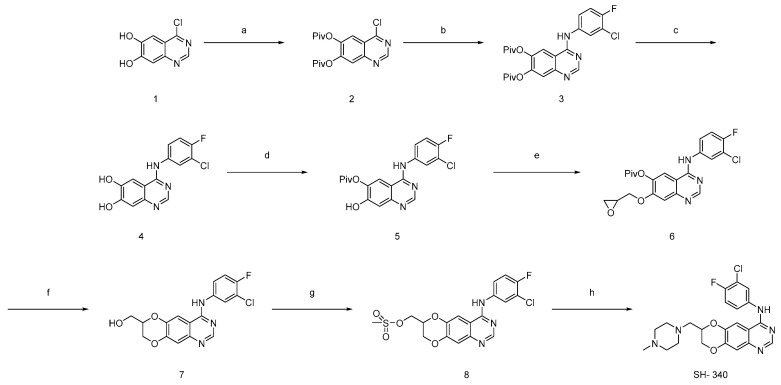
Chemical structure and synthesis of SH-340. 1. Reagents and condition: (**a**) Piv-Cl, TEA, DCM, −10 °C to r.t; (**b**) 3-chloro-4-fluoroaniline, IPA reflux 12 h; (**c**) 7N NH_3_ in MeOH, 0–23 °C 12 h; (**d**) Piv-Cl, Et_3_N, THF −40 °C 2 h, (regioselective acylation gives 5 major product) (**e**) oxiran-2-ylmethyl 4-nitrobenzenesulfonate, K_2_CO_3_, DMF, 0 °C-rt, 12 h; (**f**) K_2_CO_3_, MeOH 12 h, rt; (**g**) Et_3_N, MsCl, THF 0–23 °C, 16 h; and (**h**) 1-methylpiperazine, Et_3_N, DMF 85 °C 24 h.

Despite the existence of a quinazoline-based drug for AD, the development of new quinazoline derivatives with improved efficacy and fewer side effects is required to enhance the existing treatments for AD. Given the limited efficacy of current treatments for atopic dermatitis, we wanted to explore novel compounds to develop more effective and targeted therapies. Here, we synthesized a quinazoline derivative SH-340, the synthesis method is shown in Figure 1, refs. [19,20,21,22,23] N-(3-chloro-4-fluorophenyl)-7-((4-methyl piperazin-1-yl)methyl)-7,8-dihydro [1,4]dioxino [2,3-g]quinazolin-4-amine), and investigated biological activities in human primary keratinocytes using in vitro AD model.

## 2. Results

### 2.1. Synthesis and Functionalization of Novel Quinazoline Compound SH-340 via Multi-Step Reactions

Commercially purchased diol (1) was subsequently protected with pivaloyl groups. We found that the use of pivaloyl groups over acetyl-protecting groups was essential in aiding the purification of the subsequent chloride (2), and the substitution of aromatic halides with nitrogen nucleophiles, 3-chloro-2-fluoroaniline, was achieved in excellent yield in i-PrOH at reflux. Deprotection of (3) via ammonolysis afforded the stable diol (4), Finally, regioselective protection of (5) was achieved using pivaloyl chloride in the presence of TEA at −40 °C to afford the key intermediate, and its regiochemistry was discussed in chemical synthesis 4.5; compound (5) was treated with oxiran-2-yl-methyl-3-nitrobenzenesulfonate to give corresponding epoxide (6), which transforms the ring opening of epoxides, leads to cyclization, and gives free hydroxy group (7); mesylation of the resultant free hydroxy group with MsCl/TEA gives (8); and subsequent displacement of mesylate with 1-methylpiperazine affords desired novel quinazoline compound SH-340.

### 2.2. SH-340 Inhibits Proliferation of Keratinocytes

**Figure 2:**
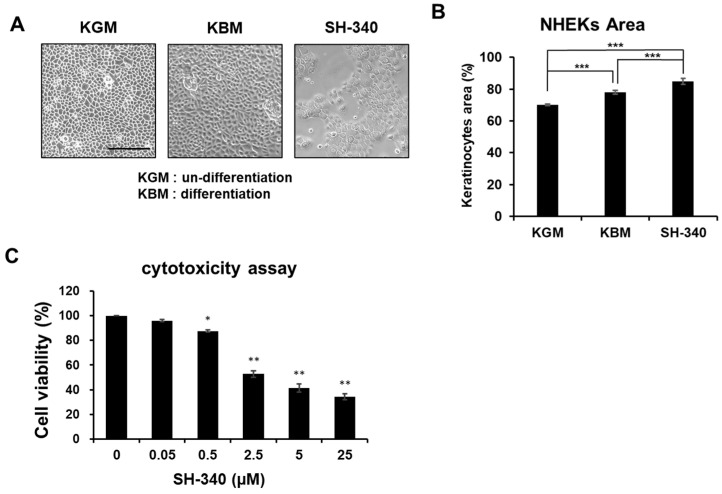
SH-340 inhibits the proliferation of keratinocytes. The morphology (**A**) and quantified cell area (**B**) of keratinocytes cultured in KGM, KBM, and KBM+SH-340 (0.5 μM). (**C**) Cell viability at different concentrations of SH-340. KGM indicates undifferentiated keratinocytes cultured in keratinocyte growth media, and KBM indicates differentiated keratinocytes cultured in keratinocyte basal media without growth factors. Scale bar: 250 μm. DMSO 1%: apoptotic positive control. (*n* = 3, *** *p* < 0.001, ** *p* < 0.01, * *p* < 0.05).

To determine the appropriate concentration of primary keratinocytes, we initially checked the cytotoxicity of SH-340 (Figure 2) at different concentrations. Cell viability results show that SH-340 has significant cytotoxicity from 0.5 to 25 μM (Figure 2C). Although keratinocytes cultured in keratinocyte growth media (KGM) maintain their stemness, they are allowed to differentiate in the basal media (KBM) without growth factors. When normal human epidermal keratinocytes (NHEKs) were treated with SH-340, we found that their growth is slow and the morphology shows a differentiated cell type with the increased cell size (Figure 2A,B). Given that the cytotoxicity assay measures the metabolic activities of mitochondria, our results indicate that SH-340 may inhibit proliferation and induce differentiation.

### 2.3. SH-340 Induces Differentiation of Keratinocytes

**Figure 3:**
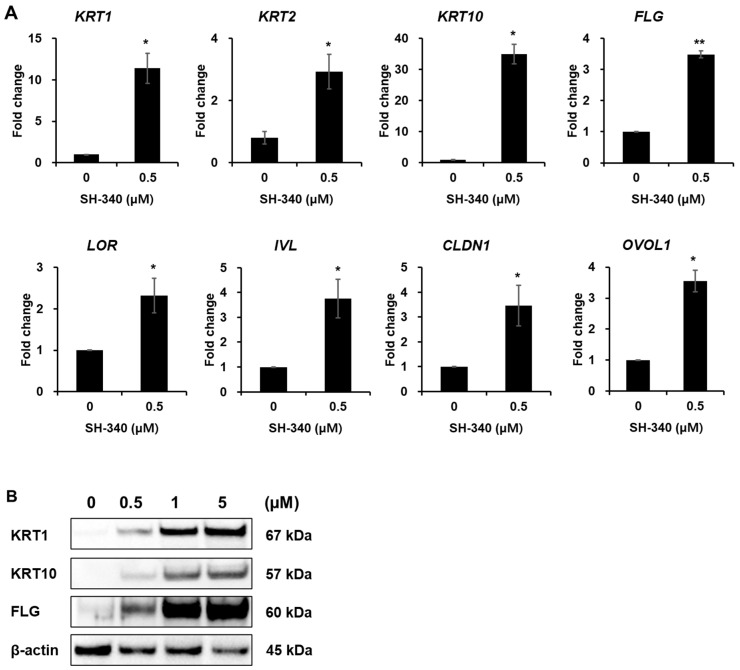
SH-340 induces the differentiation of keratinocytes. (**A**) Quantified real-time PCR results of gene expression for keratinocyte differentiation. (**B**) Western blot analysis of KRT1, KRT10, and FLG in keratinocytes treated with different concentrations of SH-340. β-actin was used as a loading control (*n* = 4–5, ** *p* < 0.01, * *p* < 0.05).

To examine whether SH-340 stimulates the differentiation of keratinocytes, we checked differentiated markers in NHEKs. NHEKs treated with SH-340 (0.5 μM) show remarkably increased gene expression of differentiation markers such as KRT1, KRT2, KRT2, and Ovo Like Transcriptional Repressor 1 (OVOL1) (Figure 3A). We also checked the expression of IVL, LOR, and Cloudin1 (CLDN1), and FLG is increased by SH-340 (Figure 2A). We confirmed that the protein expression of KRT1, KRT10, and FLG is increased in a dose-dependent manner in NHEK treated with SH-340 (Figure 3B). These results suggest that SH-340 is a potent material that can improve skin barrier function by effectively inducing NHEK differentiation.

### 2.4. SH-340 Inhibits IL-4/IL-13-Induced STAT6 Signaling

**Figure 4:**
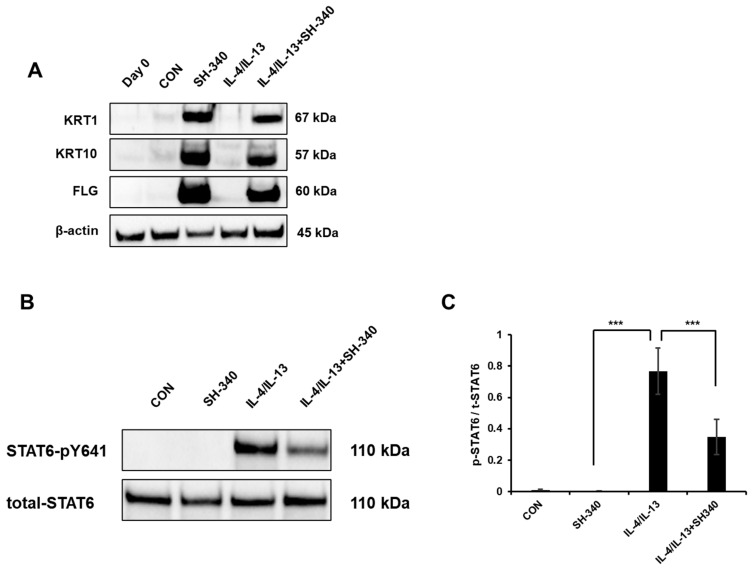
SH-340 inhibits Th2 cytokine-mediated signaling. (**A**) SH-340 restores KRT1, KRT10, and FLG expression in keratinocytes with IL-4 and IL-13. (**B**) Analysis of STAT6 phosphorylation in keratinocytes. SH-340 inhibits and significantly inhibits STAT6 phosphorylation induced by IL-4 and IL-13. (**C**) Quantified results of STAT6 phosphorylation (*n* = 6, *** *p* < 0.001).

AD is characterized by overexpression of Th2 cytokines interleukin-4 (IL-4) and IL-13, which leads to defective keratinocyte differentiation [24]. Strong research evidence proved that Th2-mediated IL-4/IL-13 inhibits gene expression of FLG by activating the JAK-STAT pathway, resulting in disruption of the skin barrier [14,25]. Thus, we attempted to verify if SH-340 regulates the IL-4/IL-13-induced signaling pathway. We identified that SH-340 induces KRT1, KRT10, and FLG protein expression (Figure 4A). Moreover, SH-340 significantly restores their expression in the presence of IL-4/IL-13 (Figure 4A). Moreover, SH-340 showed a 45% inhibition of IL-4/IL-13-induced STAT6 phosphorylation in NHEKs (Figure 4B,C). Thus, these results indicate that SH-340 has the potential to counteract the negative effects of IL-4 and IL-13 on keratinocyte differentiation and skin barrier function.

### 2.5. SH-340 Inhibits TSLP Expression in NHEK

**Figure 5:**
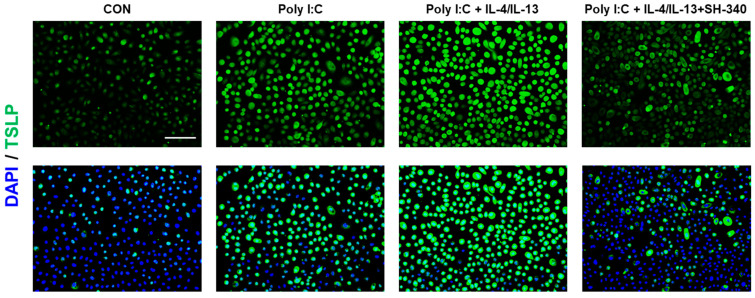
SH-340 inhibits TSLP expression induced by poly I:C or poly I:C with IL-4/IL-13 treatment. Green indicates TSLP-positive keratinocytes. Blue indicates nuclear counterstaining. The scale bar is 125 μm (*n* = 3).

The presence of IL-4 and IL-13 in skin lesions of atopic dermatitis is associated with the stimulation of thymic stromal lymphopoietin (TSLP) production in keratinocytes upon exposure to external substances [26]. Previous studies demonstrated that toll-like receptor 3 (TLR3) activation induces the expression of TSLP in human keratinocytes [27]. In addition, damaged tissues release non-coding double-stranded RNAs (dsRNAs) to activate TLR3, resulting in enhanced wound healing and tissue regeneration [28,29]. Given the repeated scratching that occurs in the skin of patients with AD, we were interested in investigating whether exposure of keratinocytes to a synthetic dsRNA (polyinosinic-polycytidylic acid; poly I:C), IL-4, and IL-13 can lead to the production of TSLP. Compared to the control group, poly I:C induced TSLP expression in keratinocytes (Figure 5). Moreover, keratinocytes exposed to poly I:C, IL-4, and IL-13 produce TSLP more than poly I:C alone (Figure 5). However, SH-340 significantly suppressed TSLP production in keratinocytes treated with poly I:C, IL-4, and IL-13 (Figure 5), suggesting that SH-340 may have the potential to alleviate pruritus caused by AD.

## 3. Discussion

Quinazoline derivatives have been studied and used in various research areas, including cancer research, neuropharmacology, inflammatory diseases, and infectious diseases. Especially, JTE-052 is a quinazoline-based drug currently being studied for the treatment of inflammatory skin diseases such as AD [18]. JTE-052 is a potent JAK inhibitor, which has been extensively studied in the context of various inflammatory disorders. Despite the existence of a few quinazoline-based drugs for AD, there are some significant reasons why we still need to study and develop quinazoline derivatives, for example, limited efficacy or safety concerns. Thus, studying and understanding the mechanisms of action of quinazoline derivatives could help researchers identify new targets and develop novel treatments for various diseases. Here, we investigated a new quinazoline molecule, SH-340, to demonstrate its potential as a therapeutic agent for AD. First, SH-340 at 0.5 μM inhibits proliferation and induces differentiation, although a high concentration of SH-340 (from 2.5 μM) showed cytotoxicity. When keratinocytes transition from basal cells to more differentiated cells, they normally change morphology, gene expression, and protein production [30,31]. The increase in cell size during keratinocyte differentiation is accompanied by the synthesis and deposition of structural proteins, which contribute to the development of a protective skin barrier. SH-340 inhibits proliferation and increases cell size, which indicates that keratinocytes may undergo a differentiation process in the presence of SH-340. We also confirmed this by analyzing differentiation factors and found that SH-340 induces keratinocyte differentiation by increasing the gene expression for initial and terminal differentiation and skin barrier functions. Second, SH-340 inhibits IL-4/IL-13-induced STAT6 signaling, which plays a key role in the pathogenesis of AD. Third, we found that SH-340 inhibits TSLP expression in NHEK, suggesting that it may attenuate the symptoms of AD by reducing the activation of dendritic cells and Th2 cells.

The potent inhibition of TSLP by SH-340 represents a significant breakthrough in immunological and therapeutic research. Since TSLP plays a pivotal role in initiating and perpetuating allergic responses, it can be a crucial target for allergic diseases such as asthma, eczema, and allergic rhinitis. The discovery that SH-340 can effectively inhibit TSLP opens up new avenues for designing innovative treatments that could attenuate allergic reactions. From a scientific standpoint, this finding deepens our comprehension of the intricate pathways underlying allergic responses, potentially paving the way for further insights into immunomodulation strategies. Clinically, SH-340′s ability to suppress TSLP suggests a potential breakthrough in treating a range of allergic diseases, offering a novel therapeutic angle that could complement or even surpass existing approaches. Although JTE-052′s JAK inhibitory activity positions it as a promising therapeutic agent in various inflammatory conditions, by targeting JAKs, key mediators of cytokine signaling, JTE-052 broadly affects multiple cytokine pathways, leading to the suppression of a wide range of inflammatory responses. However, when considering its impact on TSLP, the downstream consequences of JAK inhibition need to be evaluated. While TSLP expression is influenced by JAK-STAT signaling, JTE-052 has not been studied to demonstrate its effect on TSLP inhibition. Thus, the specific regulation of the IL-4/IL-13-STAT6 pathway by SH-340 offers a targeted approach to addressing TSLP-associated inflammation. By focusing on this specific pathway, SH-340′s inhibition of TSLP expression represents a precise strategy for curbing the initial triggers of AD. This targeted approach not only reduces the risk of unintended downstream effects on unrelated cytokine networks but also highlights the importance of TSLP in the context of AD. Furthermore, as TSLP influences autoimmune diseases and certain skin conditions, SH-340 might hold promise in addressing these disorders. Moreover, the compound’s mechanism of action could be harnessed for enhancing the efficacy of cancer immunotherapies by modulating immune responses. This versatility positions SH-340 as a versatile tool for exploring new therapeutic directions across diverse medical fields, making it a significant asset in drug development and clinical research.

Taken together, our results suggest that SH-340 has potential as a novel therapeutic agent for the treatment of AD. By targeting keratinocyte differentiation, skin barrier functions, and TSLP expression, SH-340 may offer a multi-faceted approach to the treatment of AD, which could address both the inflammatory and barrier dysfunction aspects of the disease. Despite these results, the effect of SH-340 on enhancing skin barrier function is reported for the first time in this study, and the exact mechanism is not yet known. Therefore, further studies are needed to fully elucidate the mechanism of action of SH-340 and to evaluate its efficacy and safety in vivo.

## 4. Materials and Methods

### 4.1. Chemical Synthesis

General methods reagents, solvents, and starting products were acquired from commercial sources. When indicated, the reaction products were purified by “flash” chromatography on silica gel (35–70 µm) with the indicated solvent system. HRMS analysis was conducted in EI mode, 70 eV, resolution 5000 using a JEOL JMS-700 high-resolution mass spectrometer (MS), LC-Ms were analyzed by ESI^+^ represented as *m*/*z* (M + H)^+^, NMR spectra were recorded in CDCl_3_ at 300 MHz, 400 MHz, 500 MHz (1H), and 101 MHz (13C), and chemical shifts are reported in δ values downfield from TMS or relative to residual chloroform (7.26 ppm, 77.0 ppm) or residual DMSO (2.50 ppm) as an internal standard. Data are reported in the following manner: chemical shift, multiplicity, coupling constant (J) in hertz (Hz), and integrated intensity and assignment (when possible). Multiplicities are reported using the following abbreviations: s, singlet; d, doublet; dd, doublet of doublets; t, triplet; q, quadruplet; m, multiplet; br s, broad signal; and app, apparent. Assignments and stereochemical determinations are given only when they are derived from definitive dimensional NMR experiments (g-HSQC) (Supplementary Materials).

### 4.2. Synthesis of 4-Chloroquinazoline-6,7-diyl bis(2,2-dimethylpropanoate) (***2***)

To a stirred suspension of commercially available 4-chloroquinazoline-6,7-diol (2.0 g, 10.17 mmol) and TEA (4.9 mL, 35.59 mmol) in dichloromethane (20 mL), trimethylacetyl chloride (Pivolyl chloride) (2.7 mL, 22.38 mmol) was added dropwise. The resulting suspension was stirred at room temperature for 2 h, distilled of excess DCM, diluted with EtOAc (500 mL), and washed with water (5 × 20 mL). The organic phase was concentrated to dryness and the residue triturated with water, and the resulting precipitate was collected by filtration, washed with water (5 × 20 mL), and dried to a constant weight to afford the title compound (2.6 g, 91%), LCMS: ESI^+^ *m*/*z* 364.82 (M + H)^+^, ^1^H NMR (300 MHz, Chloroform-*d*) δ 9.05 (s, 1H), 8.06 (s, 1H), 7.91 (s, 1H), 1.43 (d, *J* = 4.0 Hz, 18H).

### 4.3. Synthesis of 4-((3-Chloro-4-fluorophenyl)amino)quinazoline-6,7-diyl bis(2,2-dimethyl propanoate) (***3***)

A mixture of 4-chloroquinazoline-6,7-diyl bis(2,2-dimethylpropanoate) (1 g, 2.741 mmol) in *i*-PrOH (10.9 mL) was treated with 3-chloro-4-fluoroaniline (1.5 eq) and stirred at 95 °C for 12 h. When TLC showed completion of the reaction, it cooled to room temperature and evaporated to dryness. And the residue was suspended several times in diethyl ether; solidification was done and obtained a pure yellow solid compound after filtration. Obtained yield (1.2 g, 93%) LCMS: ESI^+^ *m*/*z* 474.5 (M + H)^+^, ^1^H NMR (300 MHz, Methanol-*d*_4_) δ 8.89 (s, 1H), 8.64 (s, 1H), 8.00 (dd, *J* = 6.6, 2.6 Hz, 1H), 7.88 (s, 1H), 7.71 (ddd, *J* = 8.9, 4.2, 2.7 Hz, 1H), 7.41 (t, *J* = 8.9 Hz, 1H), 1.44 (d, *J* = 9.0 Hz, 18H).

### 4.4. Synthesis of 4-((3-Chloro-4-fluorophenyl)amino)quinazoline-6,7-diol (***4***)

A stirred slurry of (1.0 g, 2.11 mmol) was treated at 0 °C, with 7 M solution of NH_3_ in MeOH (7 mL); the mixture was stirred at 0 °C for 15 min, and then at 23 °C for 4.5 h. The mixture was evaporated, and the residue was suspended in water (40 mL), stirred overnight, and filtered. The residue was washed with water (50 mL), DCM (4 × 50 mL), Et_2_O (2 × 50 mL), and dried in a desiccator to afford the title compound (0.587 g, 91%), LCMS: ESI^+^ *m*/*z* 306.7 (M + H)^+^, ^1^H NMR (300 MHz, DMSO-*d*_6_) δ 9.54 (s, 1H), 8.43 (s, 1H), 8.21 (dd, *J* = 6.9, 2.5 Hz, 1H), 7.87–7.72 (m, 1H), 7.41 (t, *J* = 9.1 Hz, 1H), 7.12 (s, 1H).

### 4.5. Synthesis of 4-((3-Chloro-4-fluorophenyl)amino)-7-hydroxyquinazolin-6-yl pivalate (***5***)

A stirred suspension of 4-((3-chloro-4-fluorophenyl)amino)quinazoline-6,7-diol (1.4 g, 4.57 mmol) in DMF (15 mL) was treated with Et_3_N (1.9 mL, 13.71 mmol), cooled to −40 °C, and treated dropwise with Piv_2_O (0.672 mL, 5.5 mmol). The mixture was stirred at −40 °C for 1 h, after which the cooling bath was removed, and stirring was continued for 2.5 h. The reaction mixture was diluted with DCM (50 mL), washed with 10% citric acid (2 × 10 mL), dried over (Na_2_SO_4_), filtered, and evaporated flash column (DCM: EtOAc 1:1) to afford solid (beige–yellow solid). Yield obtained (0.510 g, 30%) LCMS: ESI^+^ *m*/*z* 390.9 (M + H)^+^, ^1^H NMR (500 MHz, DMSO-*d*_6_) δ 11.04 (s, 1H), 9.66 (s, 1H), 8.54 (s, 1H), 8.20 (d, *J* = 5.5 Hz, 2H), 7.82 (dq, *J* = 9.7, 3.4, 2.9 Hz, 1H), 7.44 (t, *J* = 9.1 Hz, 1H), 7.18 (s, 1H), 1.38 (s, 9H).

The regiochemistry of the 6-OPiv-7-OH intermediate 6 was determined by NMR experiments. In comparison, to the parent bis-phenol 4, the proton at C-5 shifted 0.5 ppm downfield (as opposed to the C-8 proton which did not move) and a NOE was observed between the C-5 proton and the aniline N–H.

### 4.6. Synthesis of 4-((3-Chloro-4-fluorophenyl)amino)-7-(oxiran-2-ylmethoxy)quinazolin-6-yl pivalate (***6***)

For the synthesis, the following instructions should be followed: Dissolve 4-((3-chloro-4-fluorophenyl)amino)-7-hydroxyquinazolin-6-yl pivalate (0.180 g, 0.461 mmol) in N, N-dimethylformamide (2 mL) then add K_2_CO_3_ at 0 °C and stir for 10 min, then add oxiran-2-ylmethyl 4-nitrobenzenesulfonate (1.1 eq) and stir at 0 °C, for 30 min more, and bring RM to room temperature; continue stirring for 6 h. When TLC shows completion of the reaction, extract RM with EtOAc (50 mL) and wash with water (3 × 20 mL), then wash with a brine solution to remove excess DMF. This organic phase was concentrated and used in the next step without purification. Obtained yield, 0.140 mg, 68%. LCMS: ESI^+^ *m*/*z* 446.9 (M + H)^+^, ^1^H NMR (400 MHz, Methanol-*d*_4_) δ 8.53 (s, 1H), 8.10 (s, 1H), 8.06 (dd, *J* = 6.7, 2.5 Hz, 1H), 7.69 (dt, *J* = 8.8, 3.3 Hz, 1H), 7.29–7.22 (m, 2H), 4.52 (dd, *J* = 11.2, 2.1 Hz, 1H), 4.00 (dd, *J* = 11.2, 6.6 Hz, 1H), 3.37 (dd, *J* = 6.5, 4.0 Hz, 1H), 2.91 (t, *J* = 4.6 Hz, 1H), 2.78 (dd, *J* = 4.9, 2.6 Hz, 1H), 1.45 (s, 9H).

### 4.7. Synthesis of (4-((3-Chloro-4-fluorophenyl)amino)-7,8-dihydro-[1,4]dioxino[2,3-g]quinazolin-7-yl)methanol (***7***)

For the synthesis, the following instructions should be followed: Dissolve 4-((3-chloro-4-fluorophenyl)amino)-7-(oxiran-2-ylmethoxy)quinazolin-6-yl pivalate (0.230 g, 0.515 mmol) in MeOH (5 mL) then add K_2_CO_3_ (0.142 g, 1.0 mmol) and stir at rt for 12 h. When TLC shows completion of the reaction, evaporate RM to dryness, add H_2_O, adjust pH to 7 by NH_4_Cl, extract RM with EtOAc (50 mL) and wash with water (3 × 20 mL), and evaporate organic layer to dryness. There is no need to purify, by column, it can be directly used for the next step. Obtained yield (140 mg, 75%). LCMS: ESI^+^ *m*/*z* 362.85 (M + H)^+^.

### 4.8. Synthesis of (4-((3-Chloro-4-fluorophenyl)amino)-7,8-dihydro-[1,4]dioxino[2,3-g]quinazolin-7-yl)methyl methanesulfonate (***8***)

A solution of (4-((3-chloro-4-fluorophenyl)amino)-7,8-dihydro-[1,4]dioxino[2,3-g]quinazolin-7-yl)methanol (0.160 g, 0.442 mmol) in THF (10 mL) was treated with Et_3_N (1.5 eq), cooled to 0 °C, and treated dropwise with MsCl (1.3 eq). The mixture was stirred at rt for 16 h, cooled to 0 °C, treated with NaHCO_3_ (pH-7), extracted with EtOAc (50 mL), and washed with water (3 × 20 mL). The organic layer was evaporated to dryness. There was no need to purify, by column, it was directly used for the next step. Obtained yield (215 mg, 100%) LCMS: ESI^+^ *m*/*z* 440.1 (M + H)^+^.

### 4.9. Synthesis of N-(3-Chloro-4-fluorophenyl)-7-((4-methylpiperazin-1-yl)methyl)-7,8-dihydro-[1,4]dioxino[2,3-g]quinazolin-4-amine (SH-340)

Nucleophilic substitution of quinazolinyl mesylates with secondary amines: A mixture of quinazolinyl mesylate (150 mg, 0.341 mmol) in DMF (5 mL) was treated with 1-methylpiperazine (<10 eq) and Et_3_N (<10 eq), and the mixture was stirred at 85 °C for 24 h; the mixture was cooled to rt, residue was dissolved in EtOAc (50 mL) and washed with water (3 × 20 mL), excess DMF was removed by brine wash, the organic layer evaporated, and purification was conducted by 10% Mc: MeOH. Obtained yield (82 mg, 54%) LCMS: ESI^+^ *m*/*z* 444.5 (M + H)^+^ HRMS exact mass observed *m*/*z* 443.1523 with int 100%, ^1^H NMR (300 MHz, Chloroform-*d*) δ 8.63 (s, 1H), 8.02 (dd, *J* = 6.5, 2.4 Hz, 1H), 7.62–7.54 (m, 1H), 7.48 (s, 1H), 7.35 (s, 1H), 7.15 (t, *J* = 8.8 Hz, 1H), 4.48–4.38 (m, 2H), 4.13 (dd, *J* = 11.7, 8.0 Hz, 1H), 2.73 (ddt, *J* = 24.0, 15.8, 8.3 Hz, 10H), 2.42 (s, 3H). ^13^C NMR (101 MHz, CDCl_3_) δ 171.95, 156.27, 153.44, 149.04, 146.45, 143.61, 135.33, 123.77, 121.31, 121.24, 116.68, 116.46, 113.95, 110.14, 106.36, 71.61, 67.05, 62.59, 58.81, 58.14, 55.08, 54.98, 54.67, 53.57, 51.01, 46.02, 45.85, 45.56, 45.18, 41.68, 41.47.

### 4.10. Culture of Human Keratinocytes

Normal human epidermal keratinocytes (NHEK) were commercially provided from Lonza. NHEKs were grown in KGM media by adding 1 mL of KGM SingleQuots^TM^ supplements to 500 mL of KGM basal medium as per the manufacturer’s instructions. One vial of NHEKs frozen stock was thawed in a 37 °C water bath. Once thawed, the cells were transferred to a 15 mL conical tube containing 10 mL of KGM. After centrifugation at 3000 rpm for 5 min, the supernatant was removed and the cell pellet was resuspended in 2 mL of KGM. NHEKs were plated in a sterile culture flask at a density of 2 × 10^5^ cells/cm^2^ and incubated in a humidified CO_2_ incubator at 37 °C and 5% CO_2_. Media was replaced with fresh KGM every other day. Once NHEKs reached about 70–80% confluency, they were split using 0.025% trypsin-EDTA solution for a subculture. For all experiments, cells were used in passages 3–6.

### 4.11. Cytotoxicity Assay

The cytotoxicity of the extract was evaluated using the Cell Counting Kit-8 (CCK-8, Dojindo, Kumamoto, Japan) assay. Normal human epidermal keratinocytes (NHEK) were seeded at a density of 1 × 10^4^ cells/well in a 96-well plate and cultured for 24 h. After replacing the medium, the cells were treated with various concentrations (1, 2, 5, and 10 μg/mL) of the extract and incubated for 48 h in standard cell culture conditions. Next, the culture medium was supplemented with the CCK-8 solution, and the cells were further incubated for 3 h in a CO_2_ incubator. Absorbance readings were taken at 450 nm using an INNO-M microplate spectrophotometer (LTEK). The cell viability at each concentration was determined using the provided equation.

$$Cell\;Viability\;\left(\%\right)=\frac{A_{sample}}{A_{control}}\times 100$$

### 4.12. RNA Isolation and qRT-PCR

NHEKs were seeded at a concentration of 7 × 10^4^ cells/well in a 12-well plate and incubated for 24 h. After the incubation period, the cells were treated with the extract at a concentration of 1 μg/mL and further incubated for 72 h. Total RNA was extracted from the cells using the Quick-RNA Micro Prep Kit (ZYMO Research, Irvine, CA, USA), following the instructions provided by the manufacturer. The concentration and purity of the isolated RNA were assessed using a Jenway Genova Nano spectrophotometer (Cole-Parmer Ltd., Vernon Hills, IL, USA). For cDNA synthesis, the high-capacity cDNA Kit (ThermoFisher Scientific, Waltham, MA, USA) was utilized, with 1–2 μg of total RNA being used as the starting material for the reaction. For real-time quantitative polymerase chain reaction (qRT-PCR), the synthesized cDNA was prepared using a QuantStudio 3 instrument (Applied Biosystems, Carlsbad, CA, USA) and TaqMan Fast Advanced Master Mix (ThermoFisher Scientific). Specific target probes (ThermoFisher Scientific) were used, including KRT1 (Hs00196158_m1), KRT2 (Hs00166294_m1), KRT9 (Hs00413861_m1), IVL (Hs00846307_s1), FLG (Hs00856927_g1), CLDN1 (Hs00221623_m1), and TSLP (Hs00263639_m1). To normalize the results, the ribosomal protein lateral stalk subunit P0 (RPLP0, Hs00420895) was used as a loading control. The qRT-PCR conditions involved an initial incubation at 50 °C for 5 min, followed by denaturation at 95 °C for 20 s, annealing at 95 °C for 3 s, and extension at 60 °C for 20 s. A total of 40 cycles were performed. The amplified gene expression was quantitatively analyzed using the ddCt method [32] with the assistance of QuantStudio^TM^ Design & Analysis Software (version 1.5.1; Applied Biosystems).

### 4.13. Western Blot

NHEKs were plated in a 6-well plate at a density of 1 × 10^5^ cells/well and incubated for 24 h. Following the incubation period, the cells were treated with SH-340 (0.5 μM), Recombinant Human IL-4 (10 ng/mL), and IL-13 (10 ng/mL) obtained from PeproTech (Cranbury, NJ, USA) for 96 h. Subsequently, the cells were collected using a scraper, and protein lysates were prepared using RIPA lysis buffer (ThermoFisher Scientific) supplemented with protease inhibitors (GenDepot, Katy, TX, USA) and phosphatase inhibitors (GenDepot). Proteins were first extracted using RIPA buffer (#89900, ThermoFisher Scientific). NHEKs were first homogenized in ice-cold RIPA buffer containing protease and phosphatase inhibitors by sonication with 125 W and 20 kHz conditions 10 times in 2 s. The lysates were then incubated on ice for 30 min to allow complete cell lysis. After centrifugation at the maximum speed for 10 min at 4 °C, the supernatant containing the extracted proteins was collected and stored at −80 °C until further use. The protein concentration was determined using a BCA (bicinchoninic acid) assay (#23225, ThermoFisher Scientific) followed by the manual. For the experiment, protein samples were separated by electrophoresis on a polyacrylamide gel (10%) (#1610158, Bio-Rad). The separated proteins were then transferred onto the PVDF membrane (#10600021, Cytiva, Marlborough, MA, USA). The membrane was then blocked with non-fat dry milk in TBS including 0.1% Tween 20 (TBS-T) to prevent non-specific binding of the primary antibody. The primary antibody (KRT1: #sc-376224, KRT10: #sc-23877, FLG: #sc-80609, Santacruz biotechnology) was then incubated with the membrane overnight at 4 °C. After washing the membrane using TBS-T three times, a secondary antibody conjugated with horseradish peroxidase (HRP) (#31430, Invitrogen, Carlsbad, CA, USA) was added, and the membrane was incubated for 1 h at room temperature. The presence of TSLP was visualized by adding a chemiluminescent substrate using a chemiluminescent imaging system. The intensity of the signal can be quantified using densitometry. Beta-actin (#sc-47778, Santacruz biotechnology) was used as a reference protein to normalize the levels of the protein of interest.

### 4.14. Measuring of STAT6 Phosphorylation

Cells were plated in a 6-well plate with a density of 2 × 10^5^ cells/well and incubated for 24 h. Following the incubation, the cells were exposed to varying concentrations of SH-340 (0.5 μM) for a duration of 2 h. After the pre-treatment, the cells were subjected to IL-4 and IL-13 at a concentration of 10 ng/mL for 30 min to induce phosphorylation. Phosphorylation levels were assessed using Western blotting, employing phospho-STAT6 antibody (1:2000, BD Biosciences, San Jose, CA, USA) and total STAT6 antibody (1:2000, Cell Signaling, Danvers, MA, USA) through the same technique.

### 4.15. Immunostaining

To detect thymic stromal lymphopoietin (TSLP) in NHEKs, cells were seeded onto chamber slides (#154461PK, ThermoFisher Scientific). Then cells were fixed with 4% paraformaldehyde for 10 min and permeabilized with PBS including 0.4% Triton X-100 for 15 min. After washing with PBS three times, cells were blocked with PBS containing 10% normal goat serum for 1 h at room temperature. After blocking, cells were incubated with a primary TSLP antibody (1:100 dilution; Rabbit polyclonal, Cat# A13134, ABclonal, Woburn, MA, USA) in a blocking buffer overnight at 4 °C. The cells were then washed with PBS and incubated with a fluorescently labeled secondary antibody (goat anti-rabbit IgG, 1:1000 dilution, RSA1245, BioActs, Incheon, South Korea) for 1 h at room temperature. The cells were then counterstained with a nuclear stain, such as 4′,6-diamidino-2-phenylindole (DAPI) (ab104139, Abcam, Boston, MA, USA), and mounted on glass slides with a mounting medium. The cells were visualized using a fluorescence microscope (EVOS M5000 Imaging System, Invitrogen) and images were stored as JPEG files.

### 4.16. Analysis of Cell Size

To analyze the average cell size, six random regions were selected from the cell images of each group, and the area of the cell was measured using Image J software. The average values of the measured areas from three images per group were calculated, and statistical analysis was performed using Student’s *t*-test.

### 4.17. Statistics

The experiments were conducted at least three times, and the findings were presented as the mean ± standard error of the mean (SEM). Statistical significance was evaluated using Student’s *t*-test for comparing two groups and one-way ANOVA for comparing more than two groups with a significance level of *p* < 0.05.

## 5. Conclusions

In summary, the quinazoline derivatives SH-340 shows promising potential as a therapeutic for AD. Its ability to address keratinocyte differentiation, skin barrier functions, and TSLP inhibition holds implications not only for AD but also for broader allergic diseases like asthma and eczema. Additionally, SH-340’s innovative TSLP modulation offers a targeted approach to curb AD triggers without affecting unrelated pathways. Its versatility extends to autoimmune diseases and cancer immunotherapies, positioning SH-340 as a versatile tool in diverse medical fields. However, further investigation is needed to unravel its mechanisms fully, validate efficacy, and ensure safety. This study underscores SH-340’s transformative potential in dermatology and beyond, redefining AD treatment and immunotherapeutic strategies.

## Data Availability

Not applicable.

## Supplementary Materials

The following supporting information can be downloaded at: https://www.mdpi.com/article/10.3390/molecules28166119/s1, Figure S1: LC-MS analysis data, Figure S2: SH340 HRMS analysis report, Figure S3: SH-340 1H-NMR (300 MHz Chloroform), Figure S4: SH-340 13C-NMR (300 MHz Chloroform); Table S1: SH-340 LC-MS: Analytical equipment and conditions.
