# Supplementary material for: Novel Quinazoline Derivative Induces Differentiation of Keratinocytes and Enhances Skin Barrier Functions against Th2 Cytokine-Mediated Signaling
Source: Molecules. 2023 Aug 18;28(16):6119. doi: 10.3390/molecules28166119 (PMC10458605; doi:10.3390/molecules28166119)

Supplement Figures

Table S1. SH-340 LC-MS: Analytical equipment and conditions

<table>
<tr><th>Division</th><th>LC-MS(waters)</th></tr>
<tr><td>Model</td><td>Waters ACQUITY (UPLC), SQD2</td></tr>
<tr><td>Column</td><td>BEH C18 1.7 ㎛ (2.1×50 mm)</td></tr>
<tr><td>Detector</td><td>PDA Detector(ACQUITY)</td></tr>
<tr><td>Mobile phase</td><td><table>
<tr><th>time</th><th>DW(0.2 % TFA)</th><th>ACN</th><th>Flow</th><th>Curve</th></tr>
<tr><td>0</td><td>90</td><td>10</td><td>0.4</td><td>initial</td></tr>
<tr><td>0.5</td><td>90</td><td>10</td><td>0.4</td><td>6</td></tr>
<tr><td>2.0</td><td>10</td><td>90</td><td>0.4</td><td>6</td></tr>
<tr><td>2.8</td><td>10</td><td>90</td><td>0.4</td><td>6</td></tr>
<tr><td>3.5</td><td>90</td><td>10</td><td>0.4</td><td>6</td></tr>
<tr><td>5.0</td><td>90</td><td>10</td><td>0.4</td><td>6</td></tr>
</table></td></tr>
<tr><td>Flow rate</td><td>0.4 ml/min</td></tr>
</table>

Figure S1. SH-340 LC-MS Analysis Data

LC-MS Data-1	
Position: CPK-M1-015592-H03	MW= 443.91

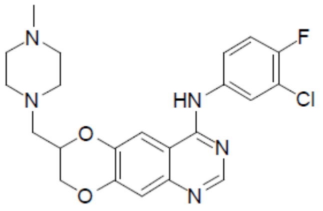

LC-MS

Sample 1 Vial 1:A,1 ID 1 File LSYA505-1 Date 07-Jan-2022 Time 09:51:09 Description

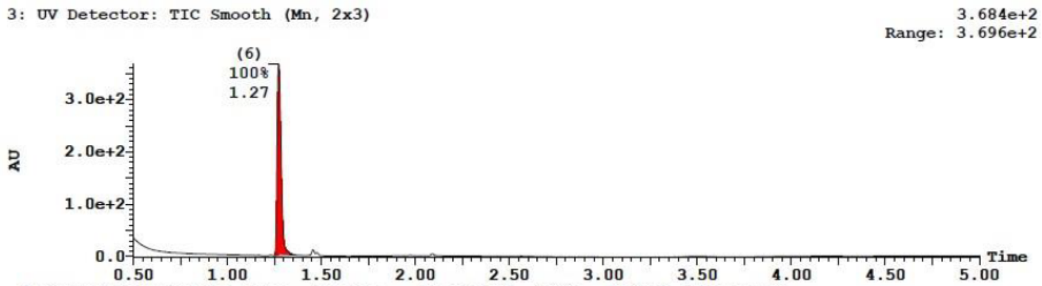

Peak Number	Compound	Time	AreaAbs	Area %Total	Width	Height	Mass Found
6		1.27	9e+006	100.00	0	4e+008	

Peak ID	Compound	Time	Mass Found
6		1.27	

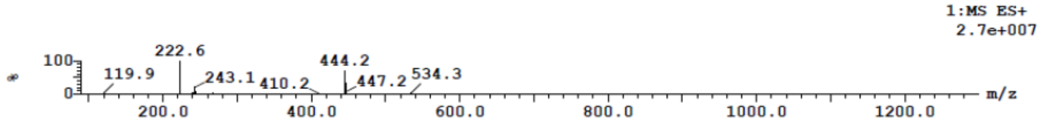

< Comment>

Position: CPK-M1-015592-H03 compound (DMSO solution)

- Peak 6 corresponds to the compound of the above structural formula and has a molecular weight (mw=444.2)

Purity: 100%

Figure S2. SH-340 HRMS analysis report

Data : 11-17-004 Date : 17-Nov-2022 17:10
Instrument : MStation
Sample : SH-430
Note :
Inlet : Direct Ion Mode : EI+
RT : 1.94 min Scan# : 59
Elements : C 22/0, H 23/0, Cl 1/0, F 1/0, N 5/0, O 2/0
Mass Tolerance : 1000ppm, 5mmu if m/z < 5, 10mmu if m/z > 10
Unsaturation (U.S.) : -0.5 - 20.0

	Observed m/z	Int%	Err[ppm / mmu]	U.S.	Composition
1	443.1523	100.00	-0.3 / -0.1	13.0	C22 H23 Cl F N5 O2

The above analysis is the result of analysis in EI mode, 70eV, Resolution 5000 using a JEOL JMS-700 High Resolution Mass Spectrometer (MS).

Figure S3. SH-340 ^{1}H-NMR (300MHz Chloroform)

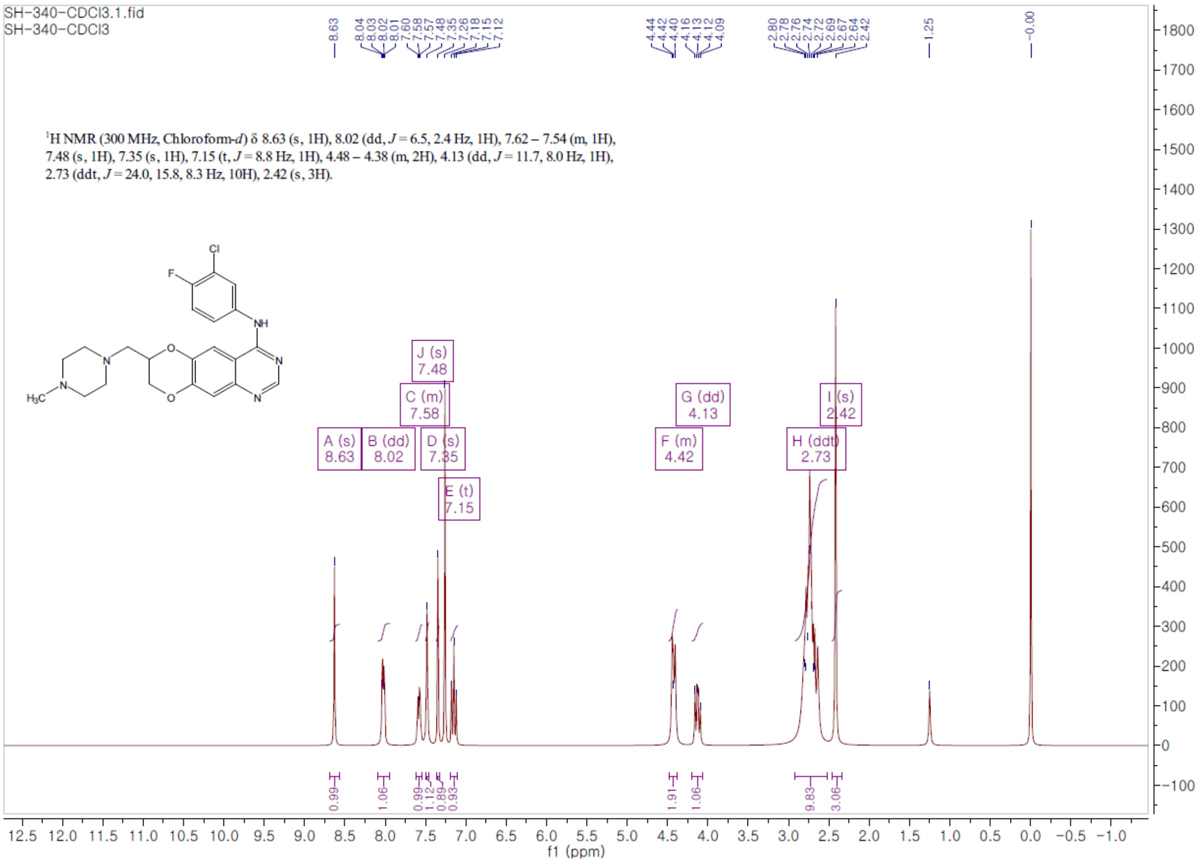

Figure S4. SH-340 ^{13}C-NMR (300MHz Chloroform)

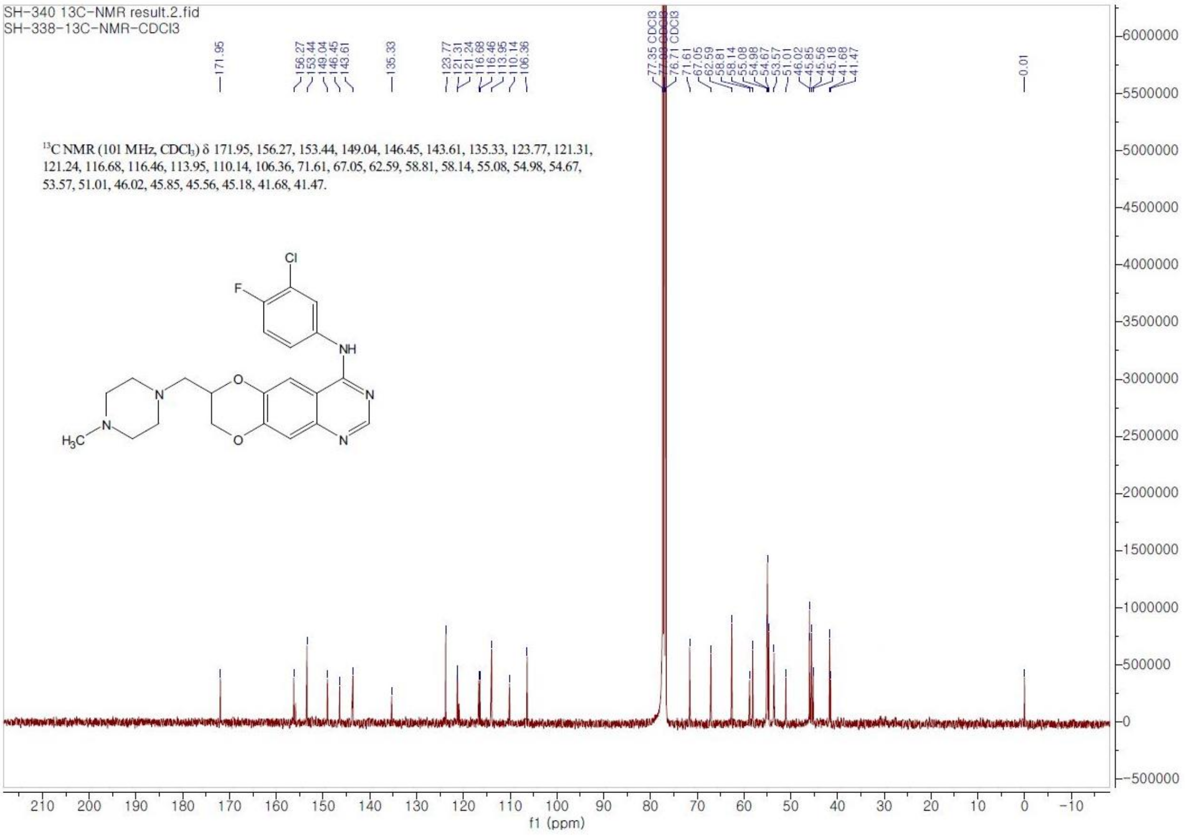

Supplement: Supplementary file 1 [file molecules-28-06119-s001.zip › molecules-2547558-supplementary.pdf]
